# MRSA: A Density-Equalizing Mapping Analysis of the Global Research Architecture

**DOI:** 10.3390/ijerph111010215

**Published:** 2014-09-30

**Authors:** Johann P. Addicks, Stefanie Uibel, Anna-Maria Jensen, Matthias Bundschuh, Doris Klingelhoefer, David A. Groneberg

**Affiliations:** 1 Division of Health Economics and Metrics, Institute of Occupational Medicine, Charité-Universitätsmedizin Berlin, Free University Berlin & Humboldt-University Berlin, Thielallee 73, D-14195 Berlin, Germany; E-Mails: jpaddicks@web.de (J.P.A.); Risikobewertung@uni-frankfurt.de (A.-M.J.); 2 Institute of Occupational Medicine, Social Medicine and Environmental Medicine, Goethe Universität Frankfurt, Theodor-Stern-Kai 7, 30590 Frankfurt, Germany; E-Mails: uibel@med.uni-frankfurt.de (S.U.); bundschuh@med.uni-frankfurt.de (M.B.); klingelhoefer@med.uni-frankfurt.de (D.K.)

**Keywords:** methicillin-resistant *Staphylococcus aureus*, MRSA, antibiotic resistance, density-equalizing mapping, scientometrics, public health

## Abstract

Methicillin-resistant *Staphylococcus aureus* (MRSA) has evolved as an alarming public health thread due to its global spread as hospital and community pathogen. Despite this role, a scientometric analysis has not been performed yet. Therefore, the NewQIS platform was used to conduct a combined density-equalizing mapping and scientometric study. As database, the Web of Science was used, and all entries between 1961 and 2007 were analyzed. In total, 7671 entries were identified. Density equalizing mapping demonstrated a distortion of the world map for the benefit of the USA as leading country with a total output of 2374 publications, followed by the UK (1030) and Japan (862). Citation rate analysis revealed Portugal as leading country with a rate of 35.47 citations per article, followed by New Zealand and Denmark. Country cooperation network analyses showed 743 collaborations with US-UK being most frequent. Network citation analyses indicated the publications that arose from the cooperation of USA and France as well as USA and Japan as the most cited (75.36 and 74.55 citations per collaboration article, respectively). The present study provides the first combined density-equalizing mapping and scientometric analysis of MRSA research. It illustrates the global MRSA research architecture. It can be assumed that this highly relevant topic for public health will achieve even greater dimensions in the future.

## 1. Introduction

First resistances against penicillin evolved in the 1940s, caused by the enzyme beta-lactamase. In the 1960s, with the use of beta-lactamase resistant antibiotics, the resistance of *Staphylococcus aureu*s against methicillin came up. Various infectious diseases are difficult to treat are due to methicillin resistant *Staphylococcus aureus* (MRSA) [1,2]. Additionally, more resistances against other antibiotics have developed, thus that hospital- or healthcare-associated MRSA (HA-MRSA) are potentially resistant to all classes of antibiotics, although individual isolates that are fully drug resistant have not been reported thus far [3]. Nevertheless, how serious the resistance against fluoroquinolone in HA-MRSA is [3] shows the use of β-lactams and fluoroquinolones that contribute to the selection of HA-MRSA clones in the hospital setting [3]. The development of the resistance mechanism seems characterized by distinct and simultaneous evolutions worldwide [4,5,6,7,8,9]. MRSA strains produce a penicillin-binding protein, PBP2a, which has a much lower affinity for beta-lactam antibiotics [10]. It appears likely that the *mec*A gene originates from similar genes found in other Gram-positive organisms, and is transferred to MRSA by horizontal genetic transfer [11].

MRSA started as a serious problem in hospitals, but, during the last few decades, it also developed into an alarming community pathogen [12], making it extremely important for public [13,14] and occupational health [15].

Despite this significance, both hospital settings and public health in general, no in-depth scientometric analysis of this important bacterium has been published thus far. Taking the overall importance of MRSA for public health in mind, the NewQIS platform (New Quality and Quantity Indices in Science) elected it as a research focus and conducted, within its focus, a combined density-equalizing mapping and scientometric analysis. NewQIS has carried out a variety of scientometric analysis of different disease patterns thus far.

## 2. Experimental Section

### 2.1. NewQIS-Platform

The present study is embedded in the NewQIS project. This project uses novel visualizing techniques in combination with scientometrics [16,17].

### 2.2. Data Source and Time Span

Data was retrieved from the Web of Science database (WoS) provided by the Thomson Institute for Scientific Information (ISI). The analysis was restricted to the period of 1900 to 2007 in order to obtain a closed period of time.

### 2.3. Search Strategies

In order to approximate the overall number of MRSA related publications the search term “MRSA*” or “methicillin-resistant Staphylococcus aureus*” or “ORSA*” or “oxacillin-resistant Staphylococcus aureus*” was entered in the search field. The Boolean operator “OR” is joining the terms together, the asterisk (*) was used to cover all possible word endings.

### 2.4. Density-Equalizing Mapping

Density-equalizing mapping was used on the basis of a method published by Gastner and Newman [18,19] of resizing territories according to a particular variable,* i.e.*, the number of published items, the average citations per item, or the h-index. The specific calculations are based on specific algorithms [18]. Publications from England, Northern Ireland, Wales and Scotland were collectively categorized as United Kingdom. Publications from “West Germany”, “Fed Rep Ger”, “Ger Dem Rep” and “Bundes Republik” were summed up as Germany. Publications from “Czechoslovakia” were assigned to either Slovakia or the Czech Republic; publications from the former “Yugoslavia” were assigned to Montenegro, Bosnia and Herzegovina, Croatia, Slovenia, Serbia or the Former Yugoslav Republic of Macedonia. Publications from “USSR” were assigned to Belarus, Ukraine, Uzbekistan, Kazakhstan, Georgia, Azerbaijan, Lithuania, Moldova, Latvia, Kyrgyzstan, Armenia, Turkmenistan, Estonia, Tajikistan or Russia.

### 2.5. Quality Criteria

For countries with at least 30 published items related to MRSA, the average number of citation per published article (citation rate) was calculated. To assess qualitative aspects of a country research activity, a country-specific h-index was established. The h-index defined by Hirsch is usually used as a quality index (*h*) for authors. An author has index *h* if *h* of his n publications have at least h citations each, and the other (*N – h*) papers have at least *h* citations each [20]. To compare the most productive countries among each other, h-Index was modified and extrapolated to the articles originated from selected countries and a country-specific modified h-index was calculated. The Impact-Factor is defined as the average number of citations per year given to those publications from an institution published in the two preceding years [21]. The Impact-Factor was used to compare the journals with the most citations among each other.

### 2.6. Analysis of International Cooperation

The author’s address was used as criterion for the publishing country in order to analyze the origin of publications. A publishing cooperation of countries could be ascertained when at least two different countries were shown in the authors’ addresses. A matrix with all identified countries was worked out and filled with the corresponding values for the cooperation of pairs of countries. A second software module was developed to translate the matrix and to transform the figures into vectors. The boldness and color of a vector corresponds to the number of cooperation-articles between the two linked countries.

### 2.7. Journal Analysis

To identify the journals with highest publication activities in the field of MRSA the set of published items was analyzed for the name of the publishing journal. In a second step, the journals were monitored for total number of citations and Impact-Factors.

## 3. Results and Discussion

### 3.1. Results

#### 3.1.1. General Parameters

**Figure 1:**
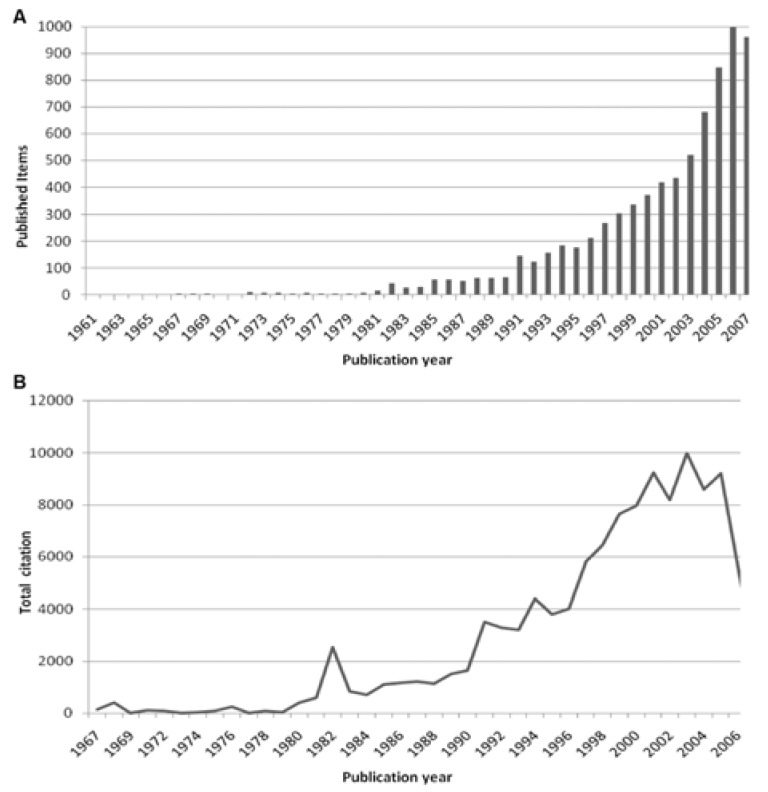
Number of publications and citations (**A**) Analysis of total number of published items. (**B**) Analysis of total number of citations of published items per year. From 1990 onwards are abstracts included to the WoS.

The total number of published items was used as an index of quantitative research productivity on the subject of MRSA. A total of 7671 items were published and included in the Web of Science database since 1961, the first year of publication until 2007. The year 2006 holds the largest number of published items (998) (Figure 1A. Concerning the total number of citation received by published items per year, the articles of 2003 rank first with a total number of 9997 citations (Figure 1B). With 17,806 citations the year 2007 is in leading position concerning the total number of citations made per year. The data illustrates a trend of an increasing number of citations both made and received since the beginning of the 1990s, which coincides with a general increase in articles published on MRSA.

#### 3.1.2. Country Research Analysis

**Figure 2:**
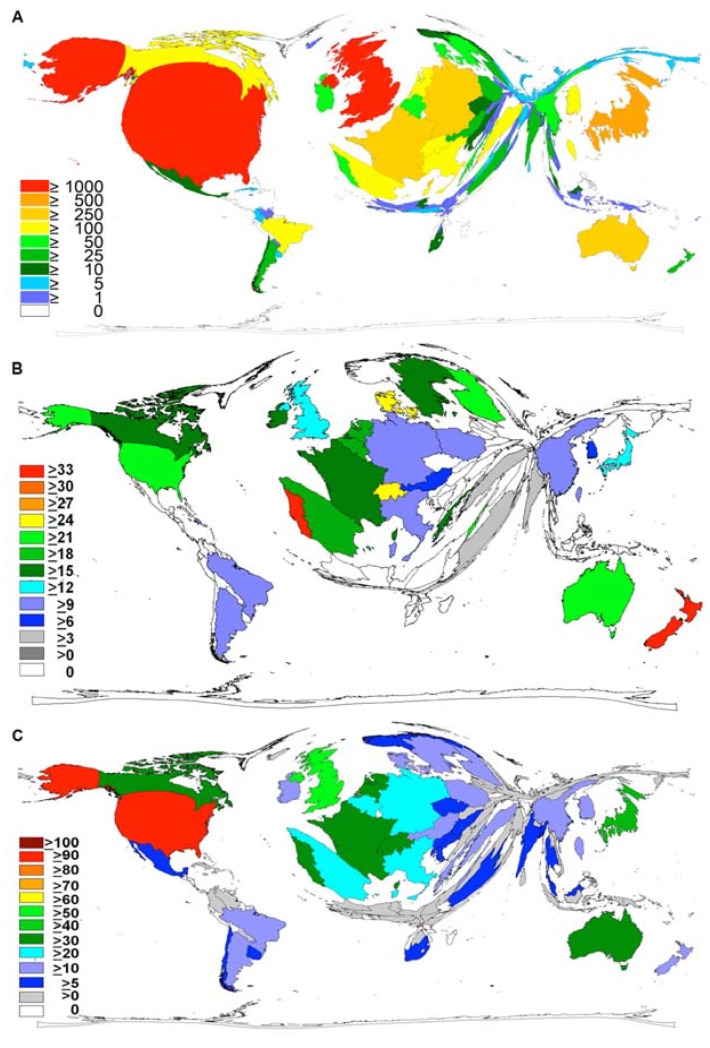
Density-equalizing calculations. (**A**) Map illustrating the number of MRSA-related articles of each country (1961–2007). (**B**) Map illustrating the citation rate of each country’s MRSA-related publications (1961–2007). (**C**) Map illustrating the country-modified h-index of each country for the period 1961–2007. In all maps, the area of each country has been scaled in proportion to the respective parameter.

Regarding the output of articles the United States of America is the most productive country with a total of 2374 publications, which is 31% of the total number of published items related to MRSA. In second position is United Kingdom with 1,030 articles (13.5%) followed by Japan with 862 (11.2%) publications. These findings are visualized by a cartogram exemplifying the data by territorial resizing (Figure 2A). When analyzing the citation rate, publications from Portugal are in the lead with 35.47 citations at an average followed by the publications from New Zealand and Denmark (Figure 2B). Concerning the country-modified h-index, the USA ranks first, with a value of 99 followed by UK with a value of 52 (Figure 2C).

#### 3.1.3. Country Research Network Analysis

**Figure 3:**
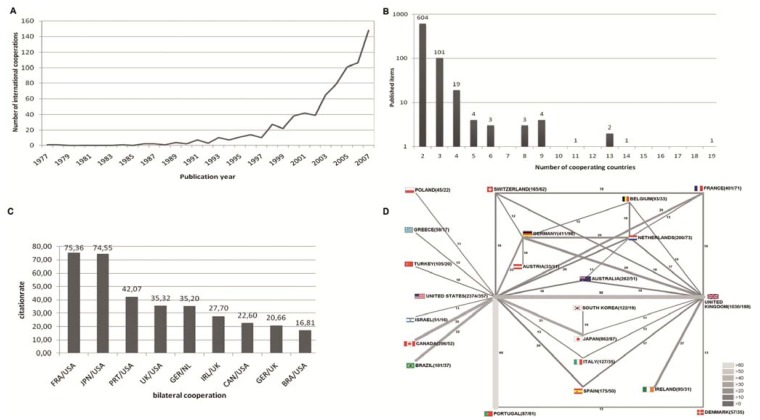
Country network analysis. (**A**) Evolution of international cooperation since 1977. (**B**) Total numbers of published items with authors originating from two, three or more countries (bi-, *tri*, and multilateral cooperation). (**C**) Bilateral cooperation with the highest citation rate. (**D**) Spider chart visualizing bilateral networking between countries for the overall number of collaborations between the two countries. Size and color of bars encode the number of bilateral cooperation. For a less crowded synoptic view a threshold of at least twelve cooperations per country for was set in the figure.

A total of 743 articles related to the topic MRSA were published as cooperation articles. The first international cooperation article was published in 1977. The years 2005–2007 represent the years with largest output of cooperation articles (Figure 3A). Six-hundred-and-four cooperation articles were published as bilateral cooperation articles. A total of 101 were the result of trilateral collaboration, 19 originated through cooperation between four countries (Figure 3B). To visualize international research networking for MRSA-related articles, the net chart technique was employed (Figure 3C). With an average citation rate of 75.36 the common scientific works of USA and France are the leading ones, close followed by the cooperations of USA and Japan. The network figure (Figure 3D) shows also the USA in the center of the cooperation works with strong connections to UK, Portugal, France, Japan and various other countries. However, there is also a broad developed network between the other countries shown.

#### 3.1.4. Journal Analysis

**Figure 4:**
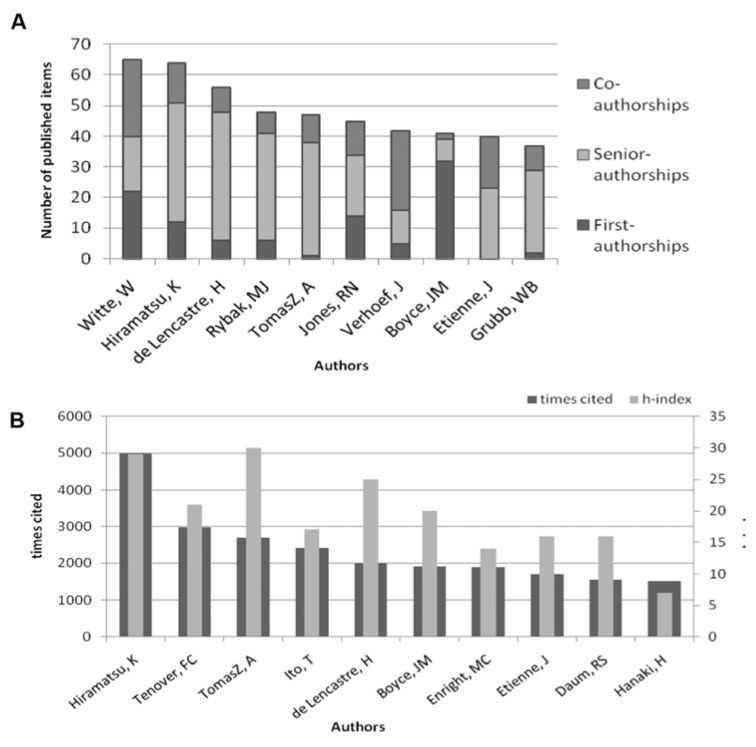
Journal analysis (**A**) The top ten journals according to total number of published items with total number of citations. (**B**) The top journals according to total number of citation with Impact-Factor.

The ten most productive journals during the period from 1961 to 2007 are ranked after their quantity of publications (Figure 4A). As a quality-indicator of the journals, the number of citation of published MRSA-related articles and Impact-Factor were used (Figure 4B). The “Journal of Hospital Infection” with 493 published items is the most productive journal regarding the topic MRSA. Further, the “Journal of Clinical Microbiology” is the most cited Journal (10,720 citations). “New England Journal of Medicine” is the Journal with the highest Impact-Factor (52.42).

### 3.2. Discussion

The present study was conducted to evaluate the MRSA research output quantitatively and qualitatively. Therefore a combined density equalizing mapping and scientometric analysis of the publications with MRSA as their subject was conducted by using the data of the WoS database. The number of publications about MRSA gradually rose from 1961 onwards, the year of the first publication, until 2007.

The USA is the leading country in the field of MRSA research. It has the highest number of publications and citations, is involved in two thirds of the most cited cooperation articles, it has the highest country-specific citation rate, and reaches the best h-Index worldwide. The tendency for a relatively small number of countries to publish the majority of items was clearly illustrated by density-equalizing mapping procedures. In a first step, we observed disproportionately high average citation rates among countries with a relatively small number of items. Therefore, we established a threshold of at least 30 publications for analysis of citation rates. The most cited publications origin from cooperations between the USA and France and between the USA and Japan with an average citation rate of 75.36 (USA/FRA) and 74.55 (USA/JAP), respectively, per publication. The most productive cooperation,* i.e.*, the one with the highest number of published articles, is the one between the USA and the UK. In Europe, in addition to a high number of cooperations with the USA, the scientific network between Belgium, the Netherlands, Germany and Switzerland is very strong. After the USA the United Kingdom is the second most productive country in MRSA-publications with the second highest h-Index. The number of authors contributing to an article has increased from three in the 1960s to more than five in 2007.

According to the total number of citations, Impact-Factor, overall number of citations and average number of citations per publication (*i.e.*, citation rate), the journal Clinical Infectious Diseases is the most important journal for MRSA. The most cited articles about MRSA have been published in the New England Journal of Medicine and Lancet. The analysis of the collected data shows that the number of citations of MRSA-articles has augmented linearly in the period examined, which corresponds to the augmenting number of publications in the same period. This leads to only a small change in the average number of citations per article and per year.

Collecting data of the individual country’s contribution to international publications constituted a problem because the nomenclature used in the databases is not always unambiguous. This influences the dependent data such as number of publications, citation rate and cooperations collected for each country, with a bigger impact on countries with a minor number of publications.

The present study is in line with data obtained for global research activity in other fields including gout [22], silicosis [23], or infectious diseases, such as influenza [24], or hepatitis B research [25]. In these studies, the ranking concerning research output starts with the US being followed by the UK, Germany or Japan. This pattern is also present when a total of over 5.5 million publications was analyzed for research activity related to 21 organ systems including brain and heart [26]. In this analysis, a uniform pattern was present with the US was ranked first in every of the 21 organ systems. Number two and three were either the UK, Japan, Germany [26].

The present pattern is also similar to research on smoking and pregnancy [27], a further topic which is highly relevant for public health [28].

In contrast, there are also diseases in which a distinct research pattern is present, Namely, these diseases are neglected tropical disease such as yellow fever [29]. Here, a recent NewQIS study identified a total of 5053 yellow fever-associated publications, which were published by 79 countries. The United States (USA) was shown to have the highest publication rate at 42% (*n* = 751). However, at second place was listed Brazil (*n* = 203) as a country which is affected by this infectious disease.

From the juxtaposition of the present data with the recently published data on other diseases and organ systems, it can be summarized that MRSA seems to be a subject which is fully integrated in industrialized countries’ research focuses since the research activity pattern is similar to the common pattern of research and contrasting to the pattern found for neglected diseases.

## 4. Conclusions

The presented study is the first combined density-equalizing mapping and scientometric analysis of a microorganism which is highly relevant for public health since it targets both community and hospital settings. The data show a marked increase in research productivity during the last two decades. It can be assumed that this topic will achieve even greater research dimensions in the future.
